# An Effective Terrain Aided Navigation for Low-Cost Autonomous Underwater Vehicles

**DOI:** 10.3390/s17040680

**Published:** 2017-03-25

**Authors:** Ling Zhou, Xianghong Cheng, Yixian Zhu, Chenxi Dai, Jinbo Fu

**Affiliations:** 1 School of Instrument Science and Engineering, Southeast University, Nanjing 210096, China; s03031212@126.com (L.Z.); zhuyixian911@163.com (Y.Z.); skyype@163.com (C.D.); fjb@seu.edu.cn (J.F.); 2 Department of Physics and Electronic Engineering, Yuncheng University, Yuncheng 044000, China; 3 Key Laboratory of Micro-Inertial Instrument and Advanced Navigation Technology, Ministry of Education, Southeast University, Nanjing 210096, China

**Keywords:** tightly-coupled integration, terrain aided navigation, particle filter, autonomous underwater vehicle

## Abstract

Terrain-aided navigation is a potentially powerful solution for obtaining submerged position fixes for autonomous underwater vehicles. The application of terrain-aided navigation with high-accuracy inertial navigation systems has demonstrated meter-level navigation accuracy in sea trials. However, available sensors may be limited depending on the type of the mission. Such limitations, especially for low-grade navigation sensors, not only degrade the accuracy of traditional navigation systems, but further impact the ability to successfully employ terrain-aided navigation. To address this problem, a tightly-coupled navigation is presented to successfully estimate the critical sensor errors by incorporating raw sensor data directly into an augmented navigation system. Furthermore, three-dimensional distance errors are calculated, providing measurement updates through the particle filter for absolute and bounded position error. The development of the terrain aided navigation system is elaborated for a vehicle equipped with a non-inertial-grade strapdown inertial navigation system, a 4-beam Doppler Velocity Log range sensor and a sonar altimeter. Using experimental data for navigation performance evaluation in areas with different terrain characteristics, the experiment results further show that the proposed method can be successfully applied to the low-cost AUVs and significantly improves navigation performance.

## 1. Introduction

Autonomous underwater vehicles (AUVs) are becoming increasingly important for oceanographic research [1]. As the range of their deployable science instruments expands and their operating cost decreases, AUVs will become very popular tools for ocean exploration and studying.

Excellent navigation is critical to AUVs in mission scenarios [2,3,4]. AUV missions usually start from the surface where the vehicle can obtain a valid global positioning system (GPS) fix [5]. However, the GPS is not available underwater. The current navigation system used on the AUVs is an inertial navigation system (INS) [6]. In spite of the continuous and autonomous navigation information, INS alone accumulates unacceptable position and velocity drift over relatively short periods of time. As a result, various combinations of an INS and sufficient external aid have become popular solutions for most AUV navigation systems [7,8,9,10]. Higher performance INS systems generally use very accurate accelerometers and gyroscopes, and incorporate software for optimal sensor fusion [11,12,13]. Current research is looking into finding further sources of aiding information to either improve navigation robustness or reduce the cost of AUV systems [14,15,16,17,18,19,20,21]. This paper concentrates on the subject of terrain-aided navigation (TAN), in which bathymetric measurements are obtained by sonar sensors, and a prior terrain map is acquired from the map database.

Terrain navigation has been used in land and air applications, such as Terrain Contour Matching (TERCOM) [22] and Sandia Inertial Terrain aided Navigation (SITAN) [23], respectively. With the extension of TAN application, the variant technique has also been applied in underwater vehicles [24,25,26].

The TAN technique can be used to provide a bounded position error with accuracy dependent on the variability of the terrain and the quality of the employed topographic map and sensors. The use of a highly accurate INS allows for the accurate alignment of successive range measurements. In addition, the use of dense and information-rich ranging sensors, such as multi-beam echo sounders (MBE) [24,25], allows for both faster convergence and increased accuracy. TAN performance on sensor-rich AUVs has been evaluated in sea trials [6,27].

To achieve long range navigation with low-cost sensors, low-accuracy navigation systems require a different approach due to large sensor errors which result in highly inaccurate alignment of INS and successive sonar beams [28]. Motivated by the research of tightly-coupled integration on the BIAUV [29], we present an effective tightly-coupled TAN method for low-cost AUVs with non-inertial grade navigation sensors and fewer beams per measurement, such as a 4-beam Doppler Velocity Log (DVL) range sensor and a sonar altimeter (single-beam echo sounder, or SBE). Using experimental data to evaluate TAN performance in areas with different terrain characteristics at a map resolution of 10 m, the experiment results further demonstrate the ability of the proposed method to significantly improve the navigation performance on low-cost AUVs.

The structure of this paper is as follows: after this Introduction, Section 2 reviews related works in TAN research. Next, the tightly-coupled TAN system is proposed in Section 3. Section 4 details the system framework and simulation platform. Then the performance of the proposed approach is validated using the results of the simulation experiments in Section 5. Finally, conclusions are drawn in Section 6.

## 2. Related Works

Terrain navigation can be carried out both in batch mode and recursively. In a recursive algorithm, the estimate is being updated recursively as each measurement arrives. Many algorithms in recursive form are further classified into loosely-coupled modules and tightly-coupled modules.

Many existing TAN applications rely upon a combination of highly accurate inertial sensors and dense range sensors (with MBE). In [27], the TAN is formulated as a recursive state-space estimation, which is highly nonlinear due to the nonlinear nature of the terrain measurement function. As a consequence, nonlinear estimation methods like point mass filters (PMFs), particle filters (PFs) and sigma point Kalman filters (SPKF) must be used. Due to the computational complexity of the estimation methods, a three-dimensional state-space model has been successfully implemented on the Kongsberg HUGIN AUV. All test methods are able to estimate the position of the AUV with accuracy within the horizontal resolution of the terrain map over underlying terrain containing enough terrain information. In [6], PF estimation of TAN with a three-dimensional state-space model has been successfully deployed on the MARV AUV equipped with three different types of echo sounders (i.e., MBE, DVL and SBE).

Throughout the TAN literature on underwater applications, TAN position estimates are treated as a position-aiding sensor, just like a GPS position update. Thus, the terrain navigation updates are loosely coupled with the INS. This approach makes the terrain navigation module more portable and the overall system more robust against errors in the terrain navigation updates. High-accuracy sensors have limited the use of TAN to only high-cost AUVs. Tightly-coupled module is another system design to study the TAN performance of using different accuracy sensors, especially the TAN performance of low-accuracy sensors for low-cost AUVs.

According to the quality of the sensors employed, due to the large inaccuracy inherent in low-grade navigation systems, it is necessary to exploit the inertial states of the main navigation system in the terrain navigation algorithm. Therefore, an augmented high-dimensional state-space model must be used. In [29], a tightly-coupled TAN uses an augmented estimation state to incorporate critical sensor errors into the PF, and it is successfully employed on sensor-limited AUVs equipped with a simple 4-beam DVL range sensor. There remain many challenges for successful TAN application on low-cost AUVs, although the research of the tightly-coupled TAN has made great progress. Motivated by the research in the literature, an effective tightly-coupled method for TAN is proposed. The method estimates the critical sensor errors by incorporating raw sensor data directly into the augmented TAN system. Furthermore, three-dimensional distance errors are calculated, providing measurement updates through the PF for the absolute and bounded-error position. The development of the TAN system is elaborated for a vehicle equipped with a non-inertial-grade strapdown inertial navigation system (SINS) [30], a 4-beam DVL and a sonar altimeter. Compared with loosely-coupled TAN, the proposed method is more effective in experiment implementation on a low-cost AUV in areas with different terrain characteristics.

## 3. TAN System Models

In this section, the tightly-coupled model used in AUVs with low-grade navigation systems and bathymetric sonar is proposed. Because of the large inaccuracy inherent in low-grade navigation systems, the proposed model could be performed in an augmented high-dimensional nonlinear TAN filter.

### 3.1. State-Space Model

Considering the coordinate reference frame for surface vessel navigation, the navigation frame (*n*-frame) is a local geographic reference frame which has its origin at the location of the navigation system and its axes aligned with the directions of east, north, and the local vertical (up). The vehicle body frame (*b*-frame) is an orthogonal coordinate aligned with the pitch, roll and yaw axes of the vehicle where the navigation system is installed. The design of a TAN system for a non-inertial-grade INS should incorporate more states that are associated with the primary errors. The augmented estimation state vector for the low-cost AUV is expressed as follows:

(1) $$x_{k+1}=x_{k}+\left\{\begin{array}{l} \left[\begin{array}{ccc} 1 & 0 & 0\\ 0 & 1 & 0 \end{array}\right]C_{b}^{n}v_{k}^{b}\Delta t\\ 0_{3}\\ \left[\begin{array}{cc} -\frac{\sin\gamma_{k}}{\cos\theta_{k}} & \frac{\cos\gamma_{k}}{\cos\theta_{k}} \end{array}\right]\left\{\begin{array}{c} \omega_{nbx,k}^{b}-\varepsilon_{x,k}^{b}\\ \omega_{nbz,k}^{b}-\varepsilon_{z,k}^{b} \end{array}\right\}\Delta t\\ 0_{2} \end{array}\right\}+e_{k}$$

where

*k* is the time index;
**x** is the system state vector, denoted as:
  (2) $$x={\left[\begin{array}{cccc} P & \Theta & \varepsilon_{x}^{b} & \varepsilon_{z}^{b} \end{array}\right]}^{T}$$
  in which **P** represents the position vector of the AUV in the *n*-frame, defined by the directions of east, north and the local vertical (i.e., depth measured by a pressure sensor in the *n*-frame), in component form:
  (3) $$P={\left[\begin{array}{ccc} x_{E} & x_{N} & x_{U} \end{array}\right]}^{T}$$
  **Θ** is the orientation vector which is composed of pitch angle, roll angle and heading angle. It may be expressed as follows:
  (4) $$\Theta ={\left[\begin{array}{ccc} \theta & \gamma & \psi \end{array}\right]}^{T}$$

The direction cosine matrix, denoted here by the symbol $C_{b}^{n}$, is a 3 × 3 matrix, the columns of which represent unit vectors in body axes projected along the reference axes. $C_{b}^{n}$ is written here in component form as follows:

(5) $$C_{b}^{n}=\left[\begin{array}{ccc} \cos\gamma \cos\psi -\sin\theta \sin\gamma \sin\psi & -\cos\theta \sin\psi & \sin\gamma \cos\psi +\sin\theta \cos\gamma \sin\psi \\ \cos\gamma \sin\psi +\sin\theta \sin\gamma \cos\psi & \cos\theta \cos\psi & \sin\gamma \sin\psi -\sin\theta \cos\gamma \cos\psi \\ -\cos\theta \sin\gamma & \sin\theta & \cos\theta \cos\gamma \end{array}\right]$$

**v***^b^* represents the velocity measured by a DVL defined by the starboard velocity and forward velocity, and up velocity in the *b*-frame, in component form:

(6) $$v^{b}={\left[\begin{array}{ccc} v_{x}^{b} & v_{y}^{b} & 0 \end{array}\right]}^{T}$$

The terms $\omega_{nbx}^{b}$ and $\omega_{nbz}^{b}$ are the *x* and *z* components of the turn rate of the *b*-frame with respect to the *n*-frame; the transport rate; $\varepsilon_{x}^{b}$ and $\varepsilon_{z}^{b}$ are gyroscope biases from the vehicle’s turn rates of the gyroscopes in the *x* and *z* directions; Δ*t* is the sample time; **e** is the system noise which represents the instrument noise together with any unmodelled biases, and so we model the **e***_k_* as being normally distributed with a mean of zero and some variance, i.e., **e***_k_*~*Ɲ*(0,**Q***_k_*). **Q***_k_* is an invertible covariance matrix.Here, the superscript *n* indicates that the variable is in the *n*-frame, whereas the superscript *b* means the *b*-frame.

Under circumstances that the vehicle moves at a constant velocity, the system model of horizontal attitude (i.e., pitch angle and roll angle) may be computed by Equations (7) and (8) [31]:

(7) $$\theta =\arcsin\left(\frac{f_{y}}{g}\right)$$

(8) $$\gamma =\arcsin\left(-\frac{f_{x}}{g\cos\theta }\right)$$

where *f_x_* and *f_y_* are the specific forces as measured by accelerometers along the starboard and forward; The gravity *g* may be derived in accordance with the following equation:

(9) $$g=\frac{g_{0}}{1+x_{U}/R_{0}}$$

where:

(10) $$g_{0}=9.780318\times (1+5.3024\times 10^{-3}\sin^{2}(L)-5.9\times 10^{-6}\sin^{2}(2L))$$

(11) $$R_{0}=\sqrt{R_{N}R_{E}}$$

(12) $$R_{N}=\frac{R(1-e^{2})}{{\left(1-e^{2}\sin^{2}(L)\right)}^{1.5}}$$

(13) $$R_{E}=\frac{R}{{\left(1-e^{2}\sin^{2}(L)\right)}^{0.5}}$$

The variables *R_N_*, *R_E_*, *R*, *e* and *L* represent the meridian radius of curvature, the transverse radius of curvature, the length of the semi-major axis, the major eccentricity of the ellipsoid of the Earth, and the latitude of the AUV, respectively.

### 3.2. Measurement Model

The measurements of east and north distance and height provided by the AUV’s navigation system constitute the measurements ($\tilde{d}$):

(14) $$\tilde{d}={\left[\begin{array}{ccc} {\tilde{d}}_{E} & {\tilde{d}}_{N} & {\tilde{d}}_{U} \end{array}\right]}^{T}$$

where ${\tilde{d}}_{E}$ and ${\tilde{d}}_{N}$ are the east and north distance computed by dead reckoning from time *k* to *k* + 1, transforming the measured velocity in the *b*-frame to the distance measurement in the *n*-frame; ${\tilde{d}}_{U}$ is the terrain altitude, which is computed by projecting the measured range (***r***), using the measurement beam unit direction vector in the *b*-frame (***a***), and the orientation vector (**Θ**), denoted as follows:

(15) $${\tilde{d}}_{U}=[\begin{array}{ccc} 0 & 0 & 1 \end{array}]C_{b}^{n}ar$$

Estimates of these measurements ($\hat{d}$) are obtained from the processing system, which is:

(16) $$\hat{d}={\left[\begin{array}{ccc} {\hat{d}}_{E} & {\hat{d}}_{N} & {\hat{d}}_{U} \end{array}\right]}^{T}$$

where ${\hat{d}}_{E}$ and ${\hat{d}}_{N}$ are the estimates of east and north distance between time *k* and *k* + 1, provided by the prediction of the state at time *k* + 1 and the filter estimates of the state at time *k*; ${\hat{d}}_{U}$ is the expected altitude at the projected location of the beams in the bathymetry map, denoted as:

(17) $${\hat{d}}_{U}=h\left(\left[\begin{array}{l} x_{E}\\ x_{N} \end{array}\right]+\left[\begin{array}{ccc} 1 & 0 & 0\\ 0 & 1 & 0 \end{array}\right]C_{b}^{n}ar\right)-x_{U}$$

The bathymetric function *h*(·) denotes an approximation to the true terrain function. In order to estimate the bathymetric value at any location, an interpolation method on the bathymetry map is used.

The three-dimensional distance measurements are compared at each measurement update to generate the filter measurement differences or innovations, denoted as **δd**, where:

(18) $$\delta d=\left[\begin{array}{c} {\tilde{d}}_{E}-{\hat{d}}_{E}\\ {\tilde{d}}_{N}-{\hat{d}}_{N}\\ {\tilde{d}}_{U}-{\hat{d}}_{U} \end{array}\right]=\left[\begin{array}{c} \delta d_{E}\\ \delta d_{N}\\ \delta d_{U} \end{array}\right]$$

The three-dimensional distance difference at time *k* may be expressed in terms of states as follows:

(19) $$y_{k}=f(x_{k})+\eta_{k}$$

where ***y****_k_* is the three-dimensional distance as described in Equation (14); *f*(·) is the corresponding distance as described in Equation (16); **η** is the measurement noise associated with measurement errors, such as velocity errors, sonar range measurement errors, reference map errors and interpolation errors. For simplicity, **η***_k_* is modelled as a zero mean white noise sequence, i.e., **η***_k_*~*Ɲ*(0, **R***_k_*), where **R***_k_* is an invertible covariance matrix.

Assuming that the sonar measurement noise is uncorrelated with the map error and errors between beams are independent, the probability of the three-dimensional distance measurement at time *k* (***y****_k_*), given vehicle state (**x***_k_*) may be expressed as follows:

(20) $$\begin{array}{cc} p(y_{k}|x_{k}) & =p(\eta_{k}=y_{k}-f(x_{k}))\\ & \propto \frac{1}{{\left(2\pi \left|R\right|\right)}^{1/2}}\exp\left(\frac{-{\left[y_{k}-f(x_{k})\right]}^{T}[y_{k}-f(x_{k})]}{2\left|R\right|}\right)\\ & \propto \frac{1}{{\left(2\pi \left|R\right|\right)}^{1/2}}\exp\left(-\frac{1}{2\left|R\right|}{\left(\sqrt{{\left(\delta d_{E,k}\right)}^{2}+{\left(\delta d_{N,k}\right)}^{2}+\sum_{i=1}^{m}{\left(\delta d_{U,k}^{i}\right)}^{2}}\right)}^{2}\right) \end{array}$$

The ∝ symbol in the above equation means that the probability is not really equal to the expression on the right side, but rather the probability is directly proportional to the right side. *m* denotes the number of beams at each bathymetric measurement.

The terrain navigation problem with its highly non-linear measurement model is an example of a highly non-linear estimation problem. For this reason, the general optimal nonlinear recursive PF [32] will be introduced.

### 3.3. PF Framework

The PF is a statistical approach for estimation and often works well for highly nonlinear problems. It is invented to numerically implement the Bayesian estimator. More specifically, we randomly generate *M* state vectors called particles that are distributed according to the probability density function (pdf) *p*(***y****_k_*|**x***_k_*) as computed by Equation (20). The set of weighted particles is described as follows:

(21) $$X_{k}=\{<x_{k}^{\left(j\right)},q_{k}^{\left(j\right)}>|j=1,2,...,M\}$$

where *M* is the number of the particles; **x**^(*j*)^ are the state vectors called particles; *q*^(*j*)^ are the particle weights.

The expected estimate value ${\hat{X}}_{k}$ that we can approximate is the algebraic mean of the particles $X_{k}$. The PF algorithm is described in Algorithm 1.
**Algorithm 1. The PF algorithm**1: Algorithm PF ($X_{k-1}$,Δ**x**_*k*,*k*−1_,***y**_k_*) 2: ${\hat{X}}_{k}=X_{k}=\emptyset$ 3: *for j* = 1 *to M do* 4: *sample*
$x_{k}^{\left(j\right)}=x_{k-1}^{\left(j\right)}+\Delta x_{k,k-1}^{\left(j\right)}+e_{k-1}$ 5: $q_{k}^{\left(j\right)}=p(y_{k}|x_{k}^{\left(j\right)})q_{k-1}^{\left(j\right)}$ 6: *add*
$<x_{k}^{\left(j\right)},q_{k}^{\left(j\right)}>$
*to*
${\hat{X}}_{k}$ 7: *end for* 8: *for j* = 1 *to M do* 9: *draw j with probability* ∝ $q_{k}^{\left(j\right)}$ 10: *add*
$x_{k}^{\left(j\right)}$
*to*
$X_{k}$ 11: *end for* 12: *return*
$X_{k}$


At the beginning of the PF, we randomly generate *M* particles that are uniformly distributed. At each time step *k* = 1, 2, …, the particles are propagated to the next time step using the process equation shown in Line 4. The state vector $x_{k}^{\left(j\right)}$ is propagated on the basis of the previous state vector $x_{k-1}^{\left(j\right)}$ and known input variable quantities Δ**x**_*k*,*k*−1_, using the state-space model given in Equation (1). After the measurement is received at time *k*, the pdf *p*(***y****_k_*|**x***_k_*) is evaluated according to the nonlinear measurement equation and the pdf of the measurement noise. In Line 5, each particle $x_{k}^{\left(j\right)}$ is weighted by expressing the likelihoods obtained in Equation (20). The resampling is implemented from Line 8 to Line 11, where low-weight particles are typically replaced by copies of high-weight particles. As a result, particles usually possess many duplicates leading to the sample impoverishment. To address this, the distribution of particles should be reinitialized if the distribution area is smaller than certain parameter value.

## 4. System Framework and Simulation Platform

**Figure 1:**
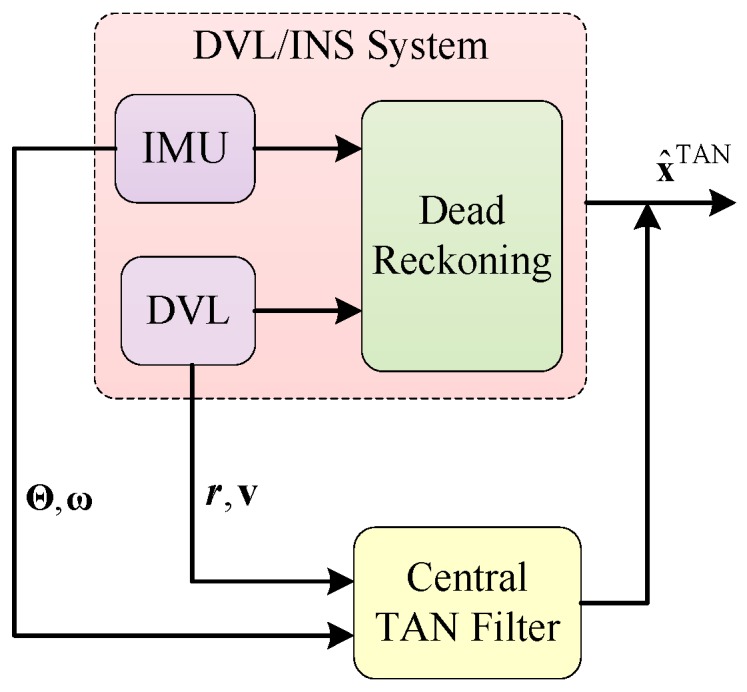
Frame diagram of the tightly-coupled integration for the TAN system.

In order to combine the data estimated by INS with the signals measured by auxiliary sensors, data fusion methods based on Bayesian filtering are used. Figure 1 displays the main functions to be implemented within a tightly-coupled TAN. The inertial data and the sonar measurements are incorporated into the TAN filter.

### 4.1. Reference Maps

A very widely used terrain map, in both underwater and terrestrial application, is the Digital Elevation Model (DEM). A typical DEM represents the terrain by a grid of elevation values, uniformly distributed in East and North. The experiments run some simulations using a prior terrain map based on real MBE data from a Marine Science Database [33]. To create a grid-based terrain map at a resolution of 10 m, the techniques of compression and smooth filter are adopted. The terrain map taken as a reference map covers a search area of around 3 km × 1.2 km, i.e., approximately 3 km from west to east and 1.2 km from south to north. The variation range of the bathymetric values is from −120.9 m to 0 m with a median of −83.3 m. Two routes are chosen in these experiments, covering different terrain to evaluate the performance of the proposed method on a low-cost AUV.

### 4.2. Reference Map and AUV Trajectory

**Figure 2:**
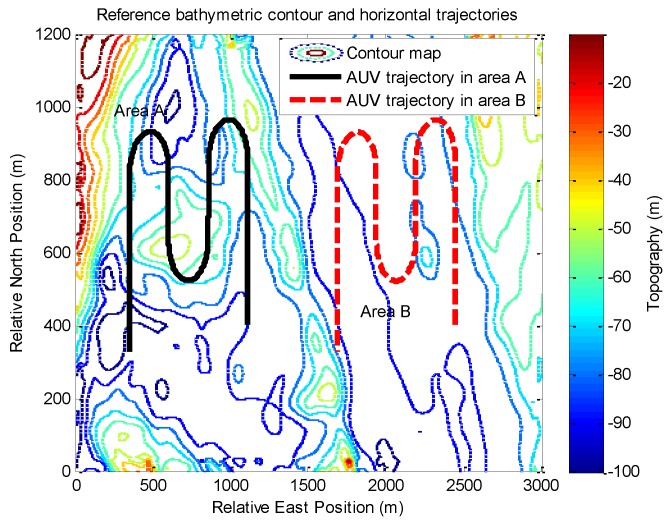
The in-water run data sets in area A and area B, shown on a bathymetric contour plot of the reference map.

To validate the results of the proposed method for the TAN with a DVL and a sonar altimeter in different terrains, Figure 2 shows the run data sets in the two areas labeled as area A and area B on a bathymetric contour plot of the reference map. Area A contains sufficient terrain variations, making it more suitable for TAN operation. On the other hand, area B is relatively flat, with significant challenges for TAN.

**Figure 3:**
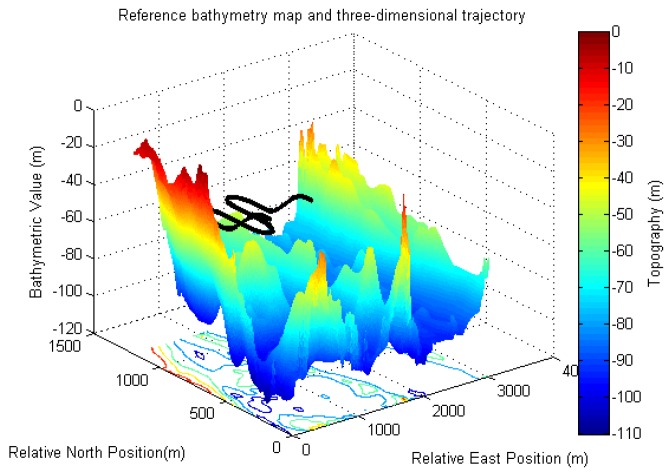
The real three-dimensional AUV trajectory in the operation area A, shown on the original reference bathymetry map at the resolution of 10 m.

The three-dimensional AUV trajectory in area A is shown in Figure 3. The AUV starts at relative east to 1111.2 m, relative north to 400.0 m and depth to −20 m. In the first 25 s, the AUV travels along a straight line to the north at the speed of 2 m/s. Then the vehicle makes a series of motions, including a crash dive for 50 s at the pitch angle rate of 0.1°/s, a crash dive for another 50 s at the pitch angle of approximately 5°, and a nose-up pitch for 50 s at the pitch angle rate of 0.1°/s. Next, the AUV enters the cruise phase traveling in a lawn-mower pattern for 840 s. In the final 175 s, the AUV travels along the opposite pitch motions to the first 175 s. The trajectory in area B is the same as that in area A, except the start position at relative east to 2444.6 m, relative north to 400.0 m and depth to −20 m.

At each of the measurement location along the AUV trajectory in area A, a set of bathymetric measurement values is distributed with a mean of around −74.7 m and a standard deviation (STD) of approximately 13.2 m. Compared to the terrain in area A, the bathymetric measurement values are distributed with a mean of around −84.2 m and some STD of roughly 4.7 m in area B. Additionally, to simulate the accumulated position errors before entering a mapped area, the AUV initial horizontal position errors are set to cover an area of 100 m × 100 m.

### 4.3. Simulation Experiment Setup

Table 1 shows a list of navigation sensors and specifications. From Table 1, the main navigation system is a non-inertial-grade SINS. Therefore, the misalignment is the primary source of navigation errors. In addition, since the particles are chosen randomly in the PF, simulation experiments using the same vehicle data will yield different results. Consequently, a series of Monte Carlo (MC) simulations are implemented for each experiment, and then the mean of the results will be treated as the final estimation. According to Table 1, Table 2 lists the experimental filter settings in MC simulations.

To recover the real AUV position estimation over large initial search area, a common choice for the initial distribution is a uniform distribution, covering a search area of 150 m × 150 m. According to the experimental conditions of sea trials [6], the reference map levels are corrected to within 0.4 m from the true value. The uncertainty of measurement quantities is at the level of ±1.0 m. In the following simulation experiments, the update period of the SINS is set to 10 ms, while the sampling intervals of the asynchronous PF filter is 1 s.

## 5. Simulation Experiment Results

Travelling over two different terrains, the AUV is equipped with the multi-sensors specified in Section 4.3. We first present navigation results obtained by the proposed method with different sonar sensors and the loosely-coupled method in rough terrain area A. To validate the ability of the proposed method on different terrains, we then perform a series of experiment on the relatively flat terrain area B. In the following section, the performances of the proposed method on the TAN will be assessed in terms of position and heading angle in detail.

**Figure 4:**
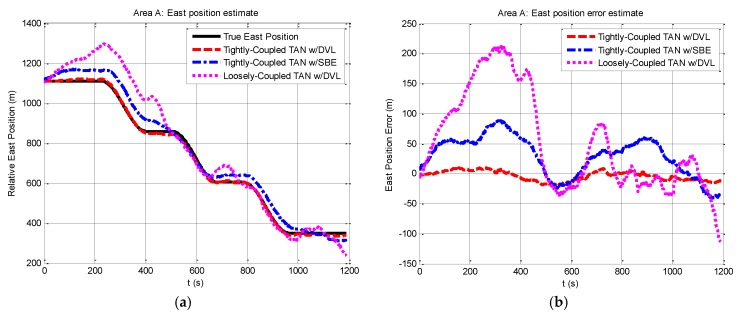
Relative east position and east position error from tightly-coupled TAN with a DVL or SBE and loosely-coupled TAN with a DVL Monte Carlo mean value over the course of the rough terrain area A run: (**a**) Relative east position; (**b**) East position error.

**Figure 5:**
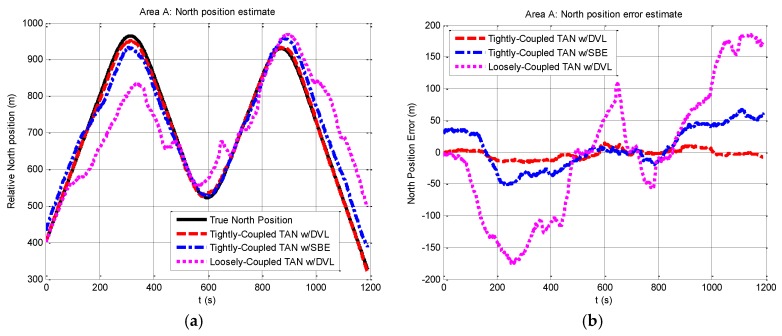
Relative north position and north position error from tightly-coupled TAN with a DVL or SBE and loosely-coupled TAN with a DVL Monte Carlo mean value over the course of the rough terrain area A run: (**a**) Relative north position; (**b**) North position error.

Navigation data estimates in area A are expressed in terms of east and north position components in Figure 4 and Figure 5. From the figures, it is clear that the performance on the tightly-coupled TAN with a DVL (red dash line) is the best to track the true position (black solid line) in both east and north position components. The estimates of the tightly-coupled TAN with a SBE for bathymetric measurements (blue dash dot line) are more accurate than the results of the traditional loosely-coupled TAN (mauve dotted line).

**Figure 6:**
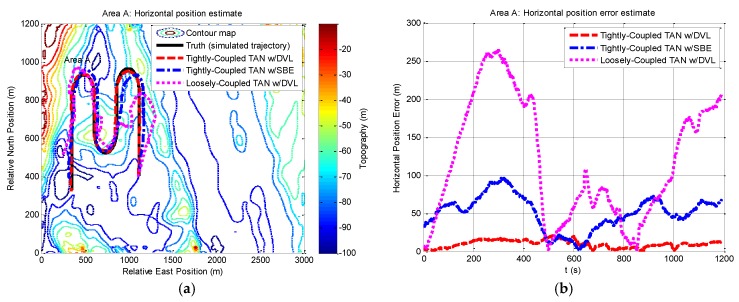
Horizontal position and horizontal position error from tightly-coupled TAN with a DVL or SBE and loosely-coupled TAN with a DVL Monte Carlo mean value over the course of the rough terrain area A run: (**a**) Horizontal position; (**b**) Horizontal position error.

Figure 6 shows two-dimensional position estimates in area A on the reference map. As shown in the figure, an estimate of the horizontal position is derived using the estimated components of position. Here we also provide statistical data in Table 3. Comparisons on the localization performances using the three methods are made in Table 3, where the performance of tightly-coupled TAN with a DVL that provides relatively more bathymetric measurements is the best in the rough terrain, achieving horizontal positioning accuracy within the map resolution. The other methods show poor performance because of less sonar beams or the algorithm itself.

**Figure 7:**
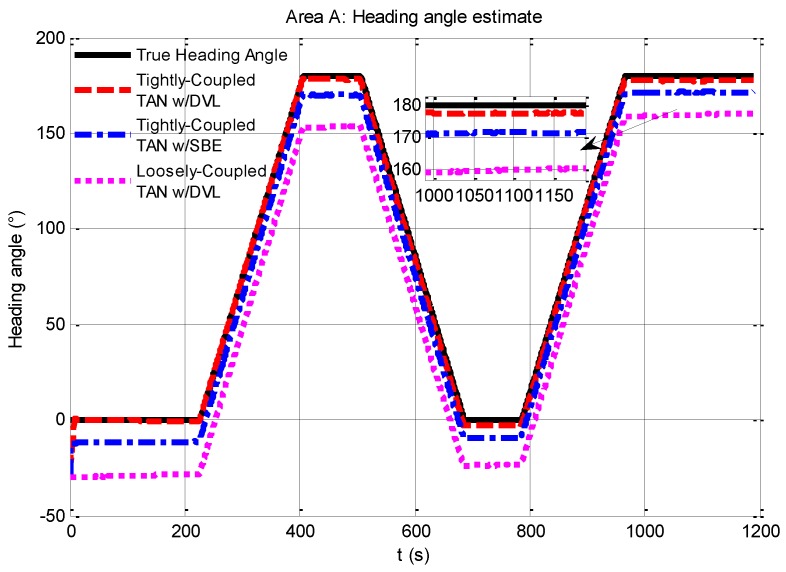
Comparison of heading angle estimates from tightly-coupled TAN with a DVL or SBE and loosely-coupled TAN with a DVL Monte Carlo mean value over the course of the rough terrain area A run.

In particular, the heading angle estimation is illustrated in Figure 7, and it is essential to study why the localization accuracy of TAN systems based on the tightly-coupled module is higher than the loosely-coupled module. As seen in Figure 7, the success of the tightly-coupled TAN is largely due to its ability to provide a significantly improved estimate of vehicle heading angle. The heading angle estimation of the tightly-coupled TAN with a DVL (red dash line) makes an improvement from the initial heading angle error of 30° down to less than 2°.

To study why the heading angle estimation of the tightly-coupled module is more accurate than that of the loosely-coupled module, the different models of the two modules should be discussed a bit closer. An augmented high-dimensional nonlinear TAN is performed in the proposed tightly-coupled TAN model by incorporating raw sensor data directly into the navigation system to estimate the critical sensor errors. Compared to the tightly-coupled TAN, TAN position estimates are treated as a position aiding sensor for the main SINS in the loosely-coupled TAN towards underwater application. The loosely-coupled TAN makes the terrain navigation module more portable and the whole integrated navigation system more efficient, while it is more difficult to exploit the inertial states of the main navigation system in the TAN. That may be the reason why the tightly-coupled TAN is more suitable for low-cost AUVs than the loosely-coupled TAN.

From the discussion of the above section, it can be seen that the TAN is able to effectively recover the correct position as long as the true position is within the search area and the proposed method could bound the position errors significantly. According to the experiment results, accurate heading angle estimation is demonstrated to be a very critical factor for the successful navigation of the low-cost AUV.

**Figure 8:**
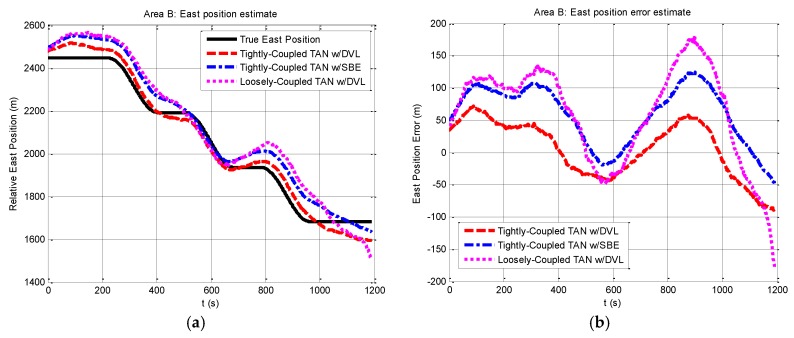
Relative east position and east position error from tightly-coupled TAN with a DVL or SBE and loosely-coupled TAN with a DVL Monte Carlo mean value over the course of the flat terrain area B run: (**a**) Relative east position; (**b**) East position error.

**Figure 9:**
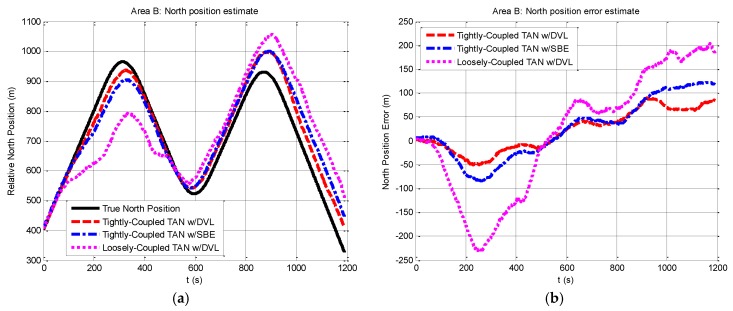
Relative north position and north position error from tightly-coupled TAN with a DVL or SBE and loosely-coupled TAN with a DVL Monte Carlo mean value over the course of the flat terrain area B run: (**a**) Relative north position; (**b**) North position error.

**Figure 10:**
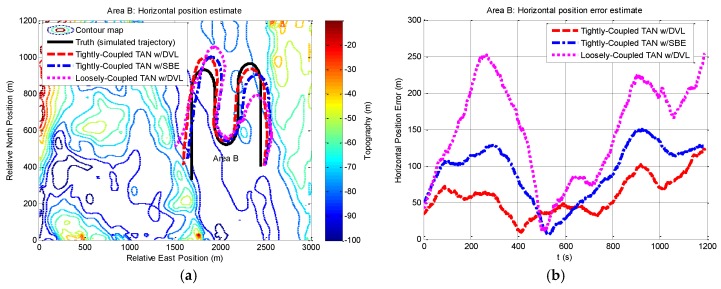
Horizontal position and horizontal position error from tightly-coupled TAN with a DVL or SBE and loosely-coupled TAN with a DVL Monte Carlo mean value over the course of the flat terrain area B run: (**a**) Horizontal position; (**b**) Horizontal position error.

We then perform a series of experiments to present navigation results obtained by the three methods in the relatively flat terrain area B. Compared with the situation in rough area A, the navigation in area B will meet more challenges due to insufficient terrain variation for TAN in the flatter area. Except the different terrain, other experimental conditions in area B are the same as those in area A. The results expressed in terms of east and north position components are shown in Figure 8 and Figure 9. From the figures, it can be seen that the tightly-coupled TAN with a DVL (red dash line) has quite equivalent performance as the tightly-coupled TAN with a SBE (blue dash dot line), which follows the true position (black solid line) in both east and north position components. Both of them are better than the position estimates from the traditional loosely-coupled TAN (mauve dotted line). In addition, Figure 10 shows a two-dimensional position estimates in area B on the reference map. As shown in the figure, the position accuracy is obviously beyond the resolution of the reference map and tends to diverge.

**Figure 11:**
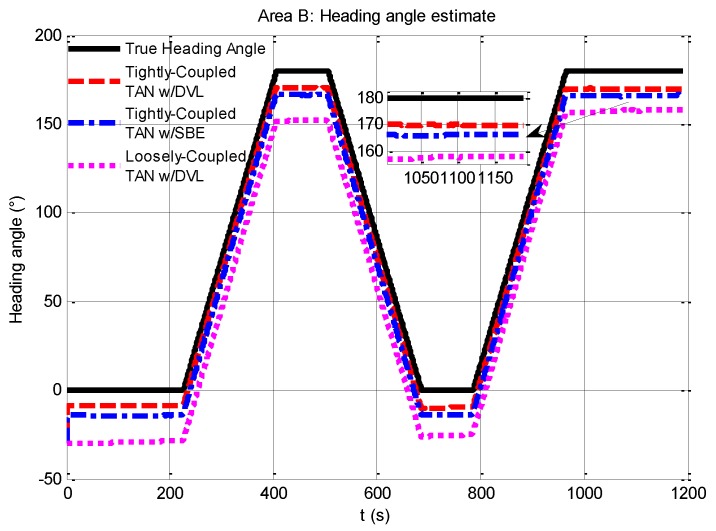
Comparison of heading angle estimates from tightly-coupled TAN with a DVL or SBE and loosely-coupled TAN with a DVL Monte Carlo mean value over the course of the flat terrain area B run.

For further analysis, Figure 11 gives an illustration of the heading angle estimation of the three methods. As seen in the figure, although the tightly-coupled TAN may provide an improved estimate of vehicle heading angle to some extent, e.g., the tightly-coupled TAN with a DVL (red dash line) can make an improvement from the initial heading angle error of 30° down to approximately 10°, the results could not meet the navigation accuracy level we would expect due to the flatness of the area.

Under the same parameter settings, the performances of the TAN by three methods on different terrains are discussed. In addition to the terrain factor, there are many system parameters affecting the TAN performance, such as the map resolutions, measurement noises, sampling time as well as number of particles. It is necessary to make more rigorous simulation studies on different system parameters. The experiments can be carried out by changing one parameter at a time while keeping all the others at their default values. Take the rough terrain area A for example, we perform a series of experiment on various system parameters to evaluate the performance of the TAN with three methods. The statistical results are shown in Table 4, Table 5, Table 6 and Table 7.

With different coarseness of the bathymetry map used in navigation, none of the three methods are able to estimate the position of the AUV with accuracy within the horizontal resolution of the terrain map, and Table 4 illustrates the deterioration of TAN performance. At the coarse maps, the position errors of the tightly-coupled methods with a DVL are almost the double of the map resolution. Comparably, the decline rate of position accuracy of the tightly-coupled method is faster than that of the loosely-coupled method. It is demonstrated that an important component of TAN performance reduction over coarser maps is decreased ability of the terrain map to represent true slope variability.

There are many factors that generate the measurement noise, as mentioned in the measurement model section. Table 5 shows the TAN performance as measurement noise changes. Compared Table 4 with Table 5, it can be seen that the changes of the TAN performance are similar in position errors and decline rate. To study why the change of different parameters leads to similar TAN performance deterioration, the relationship between the two parameters and the methods is further analyzed. In Equation (17), the accuracy of the terrain model decreases with the decline of the map resolution, in other words, it can bring about the inaccurate measurement model. Therefore, both of the two parameters affect the accuracy of the measurement model, and that may be the reason why they cause the common TAN performance change.

Sampling time is another important factor that affects the accuracy of the TAN. From Table 6, it can be seen that the TAN performance deteriorates more dramatically compared to Table 4 and Table 5. For sensor-rich AUVs, the time step between updates is usually chosen as a value that makes the distance traveled between time updates more than one map resolution to overcome the overconfidence in the estimators [6,13]. As for low-cost AUVs, the fewer time updates may be better to bound the large errors in low-accuracy sensors.

In Table 7, it shows the TAN performance at different number of particles. As well known, the number of particles is very important for PF algorithm. As usually, more particles used could achieve higher navigation accuracy to some degree, meanwhile, they will consume more time. Consequently, the number of particles will be chosen depending on the experiment.

**Figure 12:**
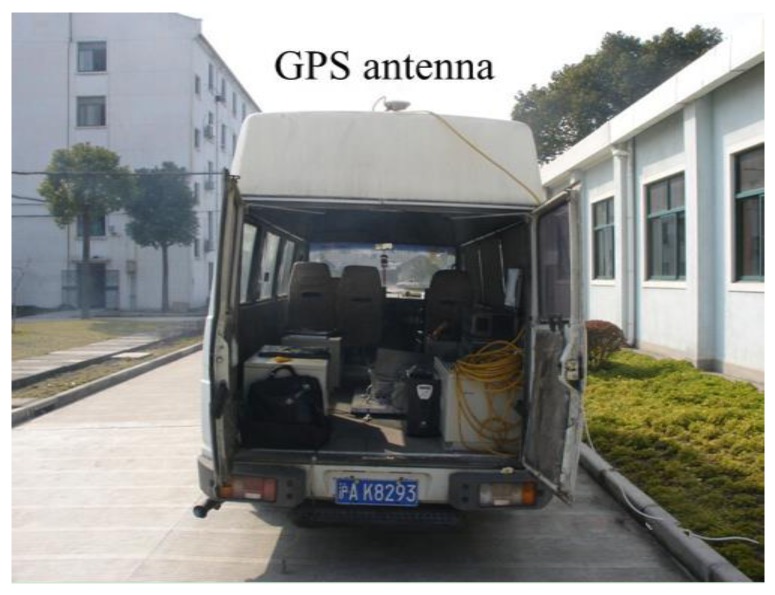
The experimental vehicle system.

**Figure 13:**
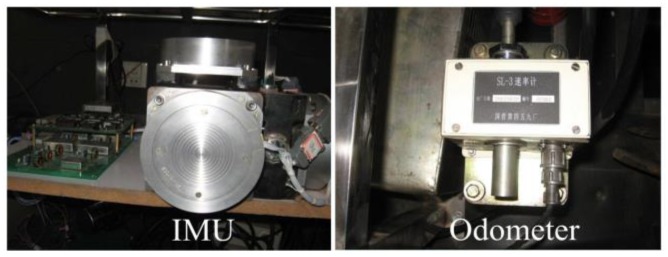
IMU and odometer.

To evaluate the proposed method further, we take the experimental data provided by the vehicle in Figure 12 as the navigation results of a vehicle on the surface of the water. The experimental vehicle system includes IMU, GPS receiver, odometer and navigation computer. The IMU and the odometer are shown in Figure 13. The reference system relies on a GPS-aided navigation-grade IMU to provide precise navigation results as reference values. The test navigation system is composed of a GPS receiver, an odometer and an IMU. The specifications of the sensors are listed in Table 8. As for the bathymetric elevation data, they are set artificially by interpolating the reference map with the resolution of 25 m. The total time of the test is 750 s.

**Figure 14:**
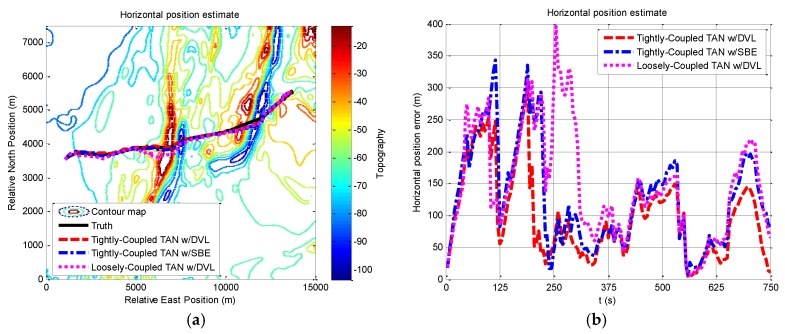
Horizontal position and horizontal position error from tightly-coupled TAN with a DVL or SBE and loosely-coupled TAN with a DVL Monte Carlo mean value over the vehicle trajectory: (**a**) Horizontal position; (**b**) Horizontal position error.

**Figure 15:**
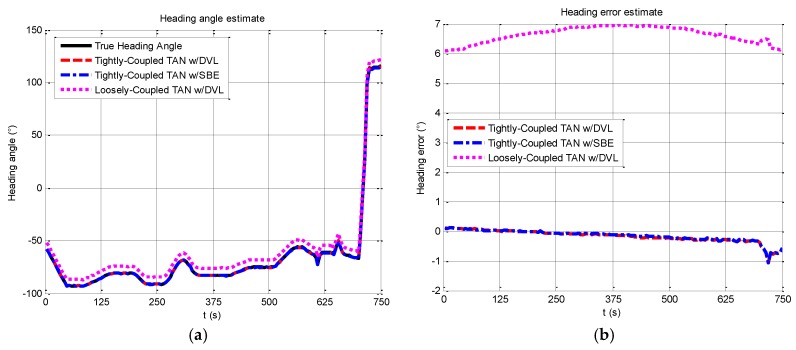
Heading angle and Heading error from tightly-coupled TAN with a DVL or SBE and loosely-coupled TAN with a DVL Monte Carlo mean value over the vehicle trajectory: (**a**) Heading angle; (**b**) Heading error.

Based on the experimental platform, the proposed method is demonstrated in the experiments. The vehicle starts from relative east at 1000.0 m, relative north at 3500.0 m in Figure 14a. Using the same trajectory on the vehicle, the performance of the proposed method on TAN is shown in terms of horizontal position and heading angle in Figure 14 and Figure 15, compared with the same module with a SBE and the loose-coupled module with a DVL. In addition, comparisons on the positioning results of the three methods are provided statistically in Table 9.

Figure 14a demonstrates the navigation performance in practice, showing different results while applying the three methods. In the test, the vehicle starts with a GPS fix, and it can be seen clearly from Figure 14b that the accuracy of the proposed method are improved between 240–750 s (rough terrain), and the tightly-coupled modules converge faster to a stable solution at around 240 s. The computational results are slightly improved from start to about 240 s (flat terrain). From statistic results in Table 9, the validity of the proposed method is demonstrated by the simulation and experimental results.

In addition to the horizontal position component, the estimates of the heading angle and heading error can be seen in Figure 15, where the proposed method succeeds in estimating the heading angle which closely follows the estimated value of the practical data, while the maximum error is about 1° at a turning around 700 s. The merit of the proposed method is its ability to provide an improved estimate of the vehicle heading.

The proposed method is validated by the results from the simulated experiments and vehicle test throughout the paper. In rough terrain area, the performance of the tightly-coupled TAN with a DVL is the best due to the tightly-coupled module, especially the significant improvement in the vehicle heading. On the other hand, the positioning results of the tightly-coupled TAN also have some improvement in the flat terrain area. The results of the simulation and the data of the experiment show consistent trends of improvement in TAN to different extent. The different accuracy is caused by practical noises existing in the corresponding test data, which will be further studied in future research.

## 6. Conclusions

In order to implement TAN for low-cost AUVs, the tightly-coupled TAN is proposed for modifications which must be applied to the navigation equations in order to take account of the large errors of low-cost AUVs, and the performance of the proposed method on the TAN is illustrated in this paper. It is clear from the simulation experiments in the preceding section that the same conclusions could be drawn from all of the experiments in different terrains: the tightly-coupled module is more suitable than the loosely-coupled module for TAN on low-cost AUVs. The statistical results of TAN localization show that the tightly-coupled TAN with a DVL that can provide relatively more bathymetric measurements performs quite well in the rough area A, with typical final errors around the map resolution, and it enables the accurate heading angle estimation, leading to the successful applications of TAN on low-cost AUVs. As a comparison, the flat area B fails to provide sufficient terrain variation for successful TAN implementation. Although the tightly-coupled TAN, compared with the loosely-coupled TAN, can also make a significant navigation improvement in relatively flat area B, it still cannot achieve accurate navigation. In addition, more rigorous simulation studies on varying system parameters are discussed. Although the TAN performance deteriorates under the adverse conditions, the results continue to be the common reference in future experiment. Finally, the proposed method is further evaluated using the test data. The comparison between the results of the simulation and the data of the experiment shows that they have good concordance.

Future studies will focus on the topic of terrain suitability for the TAN and test on the water surface using the proposed method. In addition, one of the practical limitations of TAN system is its inability without map information. A valuable extension to the limitation would be the development of SLAM technology incorporating some machine learning for bathymetric terrain model in case the map information is missing.

## Figures and Tables

**Table 1:** Experimetal sensor parameters.

Vehicle and Sensors	Variable	Specs
Vehicle	Speed	2 m/s
Gyroscopes	Initial Bias Error	±30°/h
	In-Run Bias Stability	25°/h
	Angular Random Walk	2.0°/√h
Accelerometers	Initial Bias Error	±50 mg
	In-Run Bias Stability	0.2 mg
	Noise Density	0.5 mg/√Hz
Altimeter	Altitude	2.1° beamwidth
Pressure sensor	Depth	0.01%
DVL	Range	4° beamwidth
	Velocity	±0.4% ± 0.2% cm/s

**Table 2:** Experimental filter settings.

Filter Settings	Parameters
Monte Carlo simulation settings	50
Initial convariance	diag (150 m, 150 m, 0.1 m, 0.1°/h, 0.1°/h, 30°/h, 25°/h, 25°/h)^2^
State noise convariance	diag (0.1 m/s, 0.1 m/s, 0.01, 2.0 °/√h, 2.0°/√h, 2.0°/√h, 0, 0)^2^
Measurement noise convariance	Diag (0.1, 0.1, 1)^2^ m^2^
Particle sample size	Adjustabe, with 3000 maximum
Initial area of Uncertainty	~150 by 150 m
Time step between updates	1 s
Reference map grid size	10 m

**Table 3:** Monte Carlo simulation results of the three methods final position error estimates in area A under the conditions of map resolution 10 m, measurement noise [−1 m, 1 m], sampling time 1 s, number of particles 3000.

Map Resolution (m)	Methods with Bathymetry Sensor	Mean Horizontal Position Error after 50 MC Runs (m)	Range of Final Horizontal Position Error Values after 50 MC Runs [min, max] (m)
10	Tightly-coupled w/DVL	9.7	[0.3, 20.6]
	Tightly-coupled w/SBE	50.9	[0.1, 96.1]
	Loosely-coupled w/DVL	117.9	[0.5, 266.5]

**Table 4:** Monte Carlo simulation results of the three methods final position error estimates in area A under the conditions of measurement noise [−1 m, 1 m], sampling time 1 s, number of particles 3000.

Map Resolution (m)	Methods with Bathymetry Sensor	Mean Horizontal Position Error after 50 MC Runs (m)	Range of Final Horizontal Position Error Values after 50 MC Runs [min, max] (m)
10	Tightly-coupled w/DVL	9.7	[0.3, 20.6]
	Tightly-coupled w/SBE	50.9	[0.1, 96.1]
	Loosely-coupled w/DVL	117.9	[0.5, 266.5]
20	Tightly-coupled w/DVL	31.5	[0.7, 67.3]
	Tightly-coupled w/SBE	91.5	[0.5, 259.9]
	Loosely-coupled w/DVL	144.6	[7.1, 252.8]
30	Tightly-coupled w/DVL	65.1	[0.9, 238.1]
	Tightly-coupled w/SBE	178.6	[31.6, 338.6]
	Loosely-coupled w/DVL	184.8	[2.6, 330.9]

**Table 5:** Monte Carlo simulation results of the three methods final position error estimates in area A under the conditions of map resolution 10 m, sampling time 1 s, number of particles 3000.

Measurement Noise (m)	Methods with Bathymetry Sensor	Mean Horizontal Position Error after 50 MC Runs (m)	Range of Final Horizontal Position Error Values after 50 MC Runs [min, max] (m)
[−1.0, 1.0]	Tightly-coupled w/DVL	9.7	[0.3, 20.6]
	Tightly-coupled w/SBE	50.9	[0.1, 96.1]
	Loosely-coupled w/DVL	117.9	[0.5, 266.5]
[−2.0, 2.0]	Tightly-coupled w/DVL	26.4	[0.6, 67.0]
	Tightly-coupled w/SBE	82.2	[3.5, 202.2]
	Loosely-coupled w/DVL	154.1	[5.0, 262.9]
[−3.0, 3.0]	Tightly-coupled w/DVL	64.1	[7.4, 248.3]
	Tightly-coupled w/SBE	167.7	[20.2, 343.9]
	Loosely-coupled w/DVL	175.3	[4.0, 376.7]

**Table 6:** Monte Carlo simulation results of the three methods final position error estimates in area A under the conditions of map resolution 10 m, measurement noise [−1 m, 1 m], number of particles 3000.

Sampling Time (s)	Methods with Bathymetry Sensor	Mean Horizontal Position Error after 50 MC Runs (m)	Range of Final Horizontal Position Error Values after 50 MC Runs [min, max] (m)
1	Tightly-coupled w/DVL	9.7	[0.3, 20.6]
	Tightly-coupled w/SBE	50.9	[0.1, 96.1]
	Loosely-coupled w/DVL	117.9	[0.5, 266.5]
5	Tightly-coupled w/DVL	34.5	[2.6, 71.1]
	Tightly-coupled w/SBE	98.9	[22.5, 150.2]
	Loosely-coupled w/DVL	168.3	[12.9, 272.3]
10	Tightly-coupled w/DVL	86.2	[11.3, 151.7]
	Tightly-coupled w/SBE	187.0	[15.8, 318.1]
	Loosely-coupled w/DVL	227.3	[15.5, 371.9]

**Table 7:** Monte Carlo simulation results of the three methods final position error estimates in area A under the conditions of map resolution 10 m, measurement noise [−1 m, 1 m], sampling time 1 s.

Number of Particles (s)	Methods with Bathymetry Sensor	Mean Horizontal Position Error after 50 MC Runs (m)	Range of Final Horizontal Position Error Values after 50 MC Runs [min, max] (m)
1000	Tightly-coupled w/DVL	44.9	[0.5, 128.1]
	Tightly-coupled w/SBE	71.4	[2.8, 154.0]
	Loosely-coupled w/DVL	157.6	[1.9, 269.4]
2000	Tightly-coupled w/DVL	30.5	[1.0, 72.8]
	Tightly-coupled w/SBE	61.5	[1.1, 191.7]
	Loosely-coupled w/DVL	131.6	[1.9, 220.7]
3000	Tightly-coupled w/DVL	9.7	[0.3, 20.6]
	Tightly-coupled w/SBE	50.9	[0.1, 96.1]
	Loosely-coupled w/DVL	117.9	[0.5, 266.5]

**Table 8:** Sensor specifications.

Sensors	Variable	Specs
Gyroscopes	Drift performance	1°/h
	Angular Random Walk	0.1°/√h
Accelerometers	Bias Error	0.2 mg
	Noise Density	0.2 mg/√Hz
GPS	Position	10 m
	Velocity	0.1 m/s

**Table 9:** Monte Carlo simulation results of the three methods final position error estimates over the vehicle run under the conditions of map resolution 25 m, measurement noise [−1 m, 1 m], sampling time 5 s, number of particles 3000.

Map Resolution (m)	Methods with Bathymetry Sensor	Mean Horizontal Position Error (m) and Range of the Errors [min, max] (m) after 50 MC Runs with Simulated Data	Range of Final Horizontal Position Error Values after 50 MC Runs [min, max] (m)
25	Tightly-coupled w/DVL	61.2; [1.9, 137.6]	73.1; [3.4, 153.9]
	Tightly-coupled w/SBE	85.5; [5.9, 187.6]	95.8; [4.9, 197.1]
	Loosely-coupled w/DVL	98.6; [5.4, 232.7]	107.2; [6.3, 250.2]
